# A comparison of visual assessment and semi-quantification for the diagnostic and prognostic use of [^18^F]flortaucipir PET in a memory clinic cohort

**DOI:** 10.1007/s00259-023-06583-9

**Published:** 2024-01-06

**Authors:** Gregory Mathoux, Cecilia Boccalini, Debora E. Peretti, Annachiara Arnone, Federica Ribaldi, Max Scheffler, Giovanni B. Frisoni, Valentina Garibotto

**Affiliations:** 1 Diagnostic Department, Division of Nuclear Medicine and Molecular Imaging, Geneva University Hospitals, University of Geneva, Geneva, Switzerland; 2 Università degli Studi Milano-Bicocca, Milano, Italy; 3 Laboratory of Neuroimaging and Innovative Molecular Tracers (NIMTlab), Faculty of Medicine, Geneva University Neurocenter, University of Geneva , Geneva, Switzerland; 4 Università Vita e Salute San Raffaele, Milano, Italy; 5 Department of Rehabilitation and Geriatrics, Memory Clinic, Geneva University and University Hospitals, Geneva, Switzerland; 6 Laboratory of Neuroimaging of Aging (LANVIE), University of Geneva, Geneva, Switzerland; 7 Division of Radiology, Geneva University Hospitals, Geneva, Switzerland; 8 CIBM Center for Biomedical Imaging, Geneva, Switzerland

**Keywords:** [^18^F]Flortaucipir PET, Tau stages, Alzheimer’s disease, Cognitive decline, Visual rating

## Abstract

**Purpose:**

[^18^F]Flortaucipir PET is a powerful diagnostic and prognostic tool for Alzheimer’s disease (AD). Tau status definition is mainly based in the literature on semi-quantitative measures while in clinical settings visual assessment is usually preferred. We compared visual assessment with established semi-quantitative measures to classify subjects and predict the risk of cognitive decline in a memory clinic population.

**Methods:**

We included 245 individuals from the Geneva Memory Clinic who underwent [^18^F]flortaucipir PET. Amyloid status was available for 207 individuals and clinical follow-up for 135. All scans were blindly evaluated by three independent raters who visually classified the scans according to Braak stages.

Standardized uptake value ratio (SUVR) values were obtained from a global meta-ROI to define tau positivity, and the Simplified Temporo-Occipital Classification (STOC) was applied to obtain semi-quantitatively tau stages. The agreement between measures was tested using Cohen’s kappa (*k*). ROC analysis and linear mixed-effects models were applied to test the diagnostic and prognostic values of tau status and stages obtained with the visual and semi-quantitative approaches.

**Results:**

We found good inter-rater reliability in the visual interpretation of tau Braak stages, independently from the rater’s expertise (*k*>0.68, *p*<0.01). A good agreement was equally found between visual and SUVR-based classifications for tau status (*k*=0.67, *p*<0.01). All tau-assessment modalities significantly discriminated amyloid-positive MCI and demented subjects from others (AUC>0.80) and amyloid-positive from negative subjects (AUC>0.85). Linear mixed-effect models showed that tau-positive individuals presented a significantly faster cognitive decline than the tau-negative group (*p*<0.01), independently from the classification method.

**Conclusion:**

Our results show that visual assessment is reliable for defining tau status and stages in a memory clinic population. The high inter-rater reliability, the substantial agreement, and the similar diagnostic and prognostic performance of visual rating and semi-quantitative methods demonstrate that [^18^F]flortaucipir PET can be robustly assessed visually in clinical practice.

**Supplementary Information:**

The online version contains supplementary material available at 10.1007/s00259-023-06583-9.

## Introduction

Alzheimer’s disease (AD) is characterized by the accumulation of abnormal proteins, including beta-amyloid (Aβ) and tau in the brain, leading to the formation of amyloid plaques and neurofibrillary tangles and then to the progressive decline of cognitive function [1].

The gold standard for diagnosing AD is the neuropathological examination of brain tissue obtained post-mortem [2]; however, biomarkers can help the diagnosis by detecting typical pathophysiological changes during lifetime. Positron emission tomography (PET) imaging with specific radiotracers allows to detect abnormal protein accumulation in the brain. Several radiotracers have been validated as Aβ-PET probes, and many have been approved for clinical use. These tracers bind specifically to Aβ, allowing for visualization and quantification of amyloid plaque burden in the brain. [^18^F]florbetapir (FBP), [^18^F]florbetaben, and [^18^F]flutemetamol (FMM) are among the most widely used Aβ-PET tracers. They can be visually evaluated with a binary (positive/negative) read or with various semi-quantitative approaches by employing tracer-specific masks, algorithms and software [3]. The centiloid scale has been introduced and is increasingly being adopted in clinical research to help harmonization across the various available Aβ radiotracers [4]. Tau imaging is the newest addition to PET imaging allowing for the assessment of aggregated tau in the brain [5]. [^18^F]Flortaucipir is the most widely used tracer binding with high affinity to paired helical filament tau and the only one already approved for clinical use. [^18^F]Flortaucipir PET uptake showed high correlations with neuropathology findings [6]. [^18^F]Flortaucipir PET can strongly predict cognitive changes in the preclinical and prodromal stages of AD [7, 8], overperforming Aβ-PET, FDG-PET, and structural MRI in direct comparisons [8, 9]. Currently, available research data rely mainly on semi-quantitative measures, whereas visual interpretation methods are needed for clinical translation. The accuracy of semi-quantitative metrics can be influenced by multiple factors, such as partial volume effects, threshold effects, and spillover from off-target areas [10]. Moreover, even if semi-quantitative strategies are largely used as support to the visual assessment of PET imaging in clinical routine, relying solely on semi-quantitative strategies would represent a limitation for a clinical implementation, given the variety of existing software and approaches, differing in terms of reference space, normalization strategies, specific set of regions and training datasets and the need of cross-validation [11]. Visual methods for the qualitative interpretation of [^18^F]flortaucipir PET scans have been developed and validated through autopsy studies [6] or against some semi-quantitative measures, clinical diagnosis, and Aβ status [12–14]. However, no previous study has compared a visual assessment with the Simplified Temporal-Occipital Classification (STOC), a semi-quantitative topographic scheme that allows to classify scans in predefined typical patterns mirroring the tau spreading in AD (stages 0, 1, 2, 3, and 4) or atypical patterns [15], and no previous study has evaluated the ability of visual assessment to estimate the risk of clinical progression over time in a prodromal sample of patients with Mild Cognitive Impairment (MCI).

Specifically, the FDA-approved method developed by Avid Radiopharmaceuticals involves identifying whether there is contiguous radiotracer uptake greater than 1.65 times the cerebellar uptake in the posterolateral temporal, occipital, or parietal/precuneus regions to define AD or negative tau patterns [6]. Beyond this binarized assessment, parallel efforts have been made to propose a visual rating based on the topographical distribution of the signal leading to the identification of four patterns (negative, mild temporal only, AD-like, and non-AD-like) [12]. However, no studies have yet tested the ability of the visual assessment to detect different AD stages, despite the prognostic value of Braak-based staging [16]. Indeed, *in vivo* Braak-based staging based on semi-quantification is a valuable tool for identifying individuals at higher risk of cognitive decline [16].

The present study aims to validate a systematic visual interpretation of [^18^F]flortaucipir PET scans in six levels based on the topographical distribution of tau pathology, according to Braak’s scheme [17] for the diagnostic and prognostic use of [^18^F]flortaucipir PET. For this purpose, we compared the so-derived visual ratings with two reference semi-quantitative measures (i) in their correlations with clinical stages and Aβ status, (ii) in their ability to distinguish AD patients from non-AD individuals, and (iii) in predicting cognitive changes over time in a memory clinic population.

## Methods

### Participants

The study involved 245 participants who sought consultation at the memory center of Geneva University Hospitals, ranging from individuals with normal cognitive function (CU) to those with MCI and dementia (DEM). Each participant underwent the standard clinical evaluation conducted at the memory center, which included assessments of clinical and neurological status, neuropsychological tests, and MRI scans [18]. Inclusion criteria for the study required participants to have undergone at least a tau-PET scan and a Mini-Mental State Examination (MMSE) within a one-year timeframe. For 227 participants a T1 MRI acquired within one year from the tau PET was also available and additionally, a subset of participants (*N*=206) also underwent amyloid-PET scans within one year of the tau-PET assessment. Follow-up MMSE assessments were available for a subgroup of 135 participants at 26.68±12.82 months.

The local Ethics Committee approved the imaging studies, which have been conducted under the principles of the Declaration of Helsinki and the International Conference on Harmonization of Good Clinical Practice. Each subject or their relatives provided voluntary written informed consent to participate in the studies.

### Imaging acquisition

MRI scanning was performed at Geneva University Hospitals’ Division of Radiology using a 3 Tesla scanner (Magnetom Skyra, Siemens Healthineers, Erlangen, Germany), equipped with a 20- or 64-channel head coil. The following acquisition parameters were used: repetition time [TR]=1810-1930 ms, echo time [TE]=2.19–2.36 ms, field of view=256 × 256 mm, flip angle=8°, slice thickness=0.9–1 mm, and matrix size=288 × 288 pixels or 256 × 230 pixels.

All PET scans were performed at the Division of Nuclear Medicine and Molecular Imaging at Geneva University Hospitals with Biograph128 mCT, Biograph128 Vision 600 Edge, Biograph40 mCT, or Biograph64 TruePoint PET scanners (Siemens Medical Solutions, Malvern, PA, USA). All scanners were thus from the same vendor and of the same generation, harmonized regarding their performance and reconstructions, and cross-calibrated.

[^18^F]Flortaucipir ([^18^F]-AV-1451) was synthesized at the Center for Radiopharmaceutical Sciences in Villigen, Switzerland, under license from the intellectual property (IP) owner (Avid subsidiary of Lilly, Philadelphia, PA, USA), and was used for the tau-PET scans. Subjects received 197±39 MBq of [^18^F]flortaucipir, with image acquisition performed 75 min after injection (acquisition time 30 min) [19]. Each emission frame was reconstructed in 6×5min frames and then averaged into a single image.

Aβ-PET images were acquired using either FBP or FMM tracers. In the case of FBP, images were obtained 50 minutes after the intravenous administration of 210±18MBq, consisting of 3 × 5minute image frames. For FMM, images were acquired 90 minutes after the intravenous administration of 166±16MBq, involving 4 × 5-minute image frames. Subsequently, the images were averaged to create a single frame lasting either 15 (FBP) or 20 (FMM) minutes.

For all tracers, data were acquired in list mode and reconstructed using 3D OSEM (Ordered Subset Expectation Maximization). The reconstruction process involved corrections for randoms, dead time, normalization, scatter, attenuation, and sensitivity. After applying motion correction, a 2-mm Gaussian filter with a full width at half maximum (FWHM) was employed. The resulting images had a matrix size of 400 × 400 and isotropic voxels measuring 1.01 mm.

### Imaging processing for semi-quantification purposes

PET processing was performed using Statistical Parametric Mapping (SPM12, Wellcome Trust Centre for Neuroimaging, London, UK), running in MATLAB R2018b version 9.5 (MathWorks Inc., Sherborn, MA, USA). MRI 3D T1 images were aligned to a reference plane passing through the anterior commissure, segmented into gray matter, white matter, and cerebrospinal fluid tissue compartments, and normalized to the Montreal Neurologic Institute (MNI) space using tissue probability maps. PET images were aligned to the subject’s respective T1 MRI scan, if available and performed within a year from PET-scan, and normalized to the MNI space using the transformation matrix that was generated during the registration of the MRI images to the standard space. Standardized uptake values (SUVR) for each PET modality were extracted in AD regions of interest (ROIs) obtained using the Automated Anatomic Labeling atlas 3 [20], as described in the following paragraphs.

A−: Aβ-PET images were categorized as either “A+” or “A−” using a semi-quantitative measure. To determine this classification, SUVR was calculated, with the whole cerebellum serving as the reference region. SUVR values were obtained from the centiloid volume-of-interest (VOI) and then transformed into centiloid units following the approach recommended by Klunk [4]. A centiloid value of 19 was used as the threshold to distinguish between “A+” and “A−” categories [21].

T−: Tau-PET images were semi-quantitatively assessed using two different methods. For both, SUVR was calculated using the cerebellar crus as a reference region.

- Global meta-ROI: global SUVR was calculated from the entorhinal cortex, lateral occipital cortex, inferior temporal cortex, and amygdala [22], composing the meta-ROI. To define the categories “T+” and “T−”, an internally validated global SUVR threshold of 1.24 was employed [23].
- Simplified Temporal-Occipital Classification (STOC): this scheme allows to classify scans in predefined typical patterns (stages 0, 1, 2, 3, and 4) based on ROIs located in medial, lateral, and superior temporal lobes and the primary visual cortex, or atypical patterns, as defined by Schwarz and colleagues [15]. For dichotomizing scans T− and T+ based on regional involvement, we defined T− as STOC stages 0–1 and T+ as stages 2–4. Scans classified as atypical were excluded from further analyses as they do not have a counterpart in other classifications.

### Tau PET visual interpretation method

Three nuclear medicine physicians, blinded to the subjects’ clinical information, independently evaluated all tau-PET scans independently. Physicians had varying levels of expertise: an expert (VG), a moderate expert (GM), and an inexpert (AA). Of note, the inexpert rater followed a brief training (2h). The other raters were experienced in the rating of tau PET, with an average of more than 5 and 1 years of experience, respectively. Anonymized data were analyzed in a random order to avoid bias by order effects.

To adjust the color scale of the scans, the predominant color in the inferior cerebellar cortex was set as the midpoint. This manual adjustment followed published recommendations [6] and considered regions with increased [^18^F]flortaucipir uptake, as well as areas with frequent off-target binding.

The visual interpretation method identified six levels based on the following recommendations:

- Scans without [^18^F]flortaucipir uptake beyond background in any brain area or limited to known off-target binding regions were considered negative.
- Scans with cortical uptake above background were classified following the regions used to define Braak staging [16, 17]. Subjects were classified as Braak I-III positive when the [^18^F]flortaucipir signal showed mild to moderate elevation in the medial temporal cortex and fusiform gyrus. Subjects were classified as Braak IV positive when [^18^F]flortaucipir fixation extended to the lateral temporal cortex. Subjects were classified as Braak V positive when [^18^F]flortaucipir fixation extended to the parietal or frontal cortex, and finally, subjects were classified as Braak VI positive when [^18^F]flortaucipir fixation extended to the motor and primary visual cortex.
- Scans with cortical uptake above background in other regions not considered by Braak were classified as non-AD and excluded from further analyses.

In cases where there was disagreement among the physicians’ readings, a consensus reading was obtained and then used for statistical analyses aiming at validating the visual classification against semi-quantitative classifications.

To dichotomize the scans based on visual assessment, in line with previously post-mortem validated data [6], T− was defined as stages 0 and I–III, and T+ was defined as stages IV–VI.

### Statistical analyses

Baseline demographics and clinical, cognitive, and biomarker differences among syndromic diagnoses were assessed using a Kruskal-Wallis rank sum test for continuous variables and a proportion test for categorical variables.

To evaluate the agreement between raters, Cohen’s kappa (*k*) was used as a measure of inter-rater agreement in the definition of Braak stages. This analysis aimed to assess the consistency of visual ratings among the different physicians.

To assess the agreement between different measures of T (visual and semi-quantitative), Cohen’s kappa (*k*) and weighted *k* were used for binary status (visual T status vs SUVR-based T status) and stages (visual Braak stages vs STOC stages), respectively.

We performed receiver-operating-characteristic (ROC) analyses to compare the discriminative power of T status obtained by the different modalities in differentiating between A+ and A− subjects and between Aβ-positive MCI/DEM cases and all other clinical cases. The resulting areas under the curve (AUCs) from different methods were compared using a De Long test for two correlated ROC curves.

To assess the prognostic value on cognitive outcomes of tau status and stages obtained from different approaches, linear mixed-effects models were applied. These models included random intercepts and slopes, with longitudinal Mini-Mental State Examination (MMSE) scores as the dependent variable. Age and gender were included as covariates in the analysis to account for their potential influence on cognitive outcomes over time. The same models were also run in a subsample using the Preclinical Alzheimer’s Cognitive Composite (PACC) as an independent variable instead of the MMSE (Supplementary Materials, S1).

All analyses were performed using R, version 4.0.2 (https://www.r-project.org/). A *p* value of 0.05 was considered the significance threshold for all analyses.

## Results

### Participant characteristics

Out of the total sample of 245 subjects, 72 were classified as CU, 126 as MCI [24], and 47 as DEM [25]. Of these, 52% were women, and the average age (standard deviation, SD) was 68.25 (4.54) years. Participants had an average (SD) baseline MMSE of 24.67 (3.02). 135 subjects had a follow-up (55.10%) after an average interval of 26.68 (12.82). Descriptive statistics are displayed in Table 1.

**Table 1:** Population characteristics

	CU (*N*=72)	MCI (*N*=126)	DEM (*N*=47)	*p*-value
Age	68(9.42)	73(7.6)	69(9.4)	0.0124
Sex (male/female)	28/44	68/58	21/26	0.335
Education	14.46(4.5)	14(3.8)	12(4.5)	0.0218
Amyloid status (A-/A+) ^#^	50/10	36/72	6/32	<0.001
MMSE	28(2.1)	26(2.4)	20(6.5)	<0.001

*Abbreviations: CU* cognitively unimpaired, *MCI* mild cognitive impairment, *DEM* dementia, *MMSE* Mini-Mental State Examination

^#^Amyloid status is based on PET, that was available for a subsample (*n*=206)

### Visual classification: inter-reader agreement and association with SUVR

Our analysis revealed good inter-rater reliability in the visual interpretation of tau Braak stages, regardless of the raters’ level of expertise. *k* values ranged from 0.68 (inexpert vs moderate-expert) and 0.69 (inexpert vs expert) to 0.77 (moderate-expert vs expert), indicating good agreement among the raters (*p*<0.001).

We observed a good agreement between the visual-based and the meta-ROI-based (global SUVR) classifications into positive and negative cases, with a *k* value of 0.67 (*p*<0.001). Similarly, there was a good agreement between visual interpretation and dichotomized classification of the STOC system, with a *k* value of 0.69 (*p*<0.001). The highest agreement was between status based on the two semi-quantitative methods (global SUVR and STOC) (*k*=0.75, *p*<0.001). Furthermore, when we considered all stages according to Braak visual staging and STOC model, we found a significant agreement between the two staging classifications (*k*_weighted_=0.73, *p*<0.001). The concordance of the different strategies across diagnostic categories are reported in Table 2.

**Table 2:** Concordance of different tau modalities in the different diagnostic categories

	Cohen’s kappa	*p* value
CU		
Tau status based on SUVR vs. visual	0.235	**0.008**
Tau status based on SUVR vs. STOC	0.606	**<0.001**
Tau status based on visual vs. STOC	0.345	**0.008**
Tau stages based on visual vs. STOC	0.31*	**<0.05**
MCI		
Tau status based on SUVR vs. visual	0.672	**<0.001**
Tau status based on SUVR vs. STOC	0.745	**<0.001**
Tau status based on visual vs. STOC	0.636	**<0.001**
Tau stages based on visual vs. STOC	0.72*	**<0.001**
DEM		
Tau status based on SUVR vs. visual	0.645	**<0.001**
Tau status based on SUVR vs. STOC	0.651	**<0.001**
Tau status based on visual vs. STOC	0.569	**<0.001**
Tau stages based on visual vs. STOC	0.64*	**<0.001**

*Abbreviations: CU* cognitively unimpaired, *MCI* mild cognitive impairment, *DEM* dementia, *STOC* Simplified Temporo-Occipital Classification

^*^For concordance between stages (visual and STOC), the weighted Cohen’s kappa is reported

### Tau across modalities, clinical diagnosis, and amyloid status

**Fig. 1:**
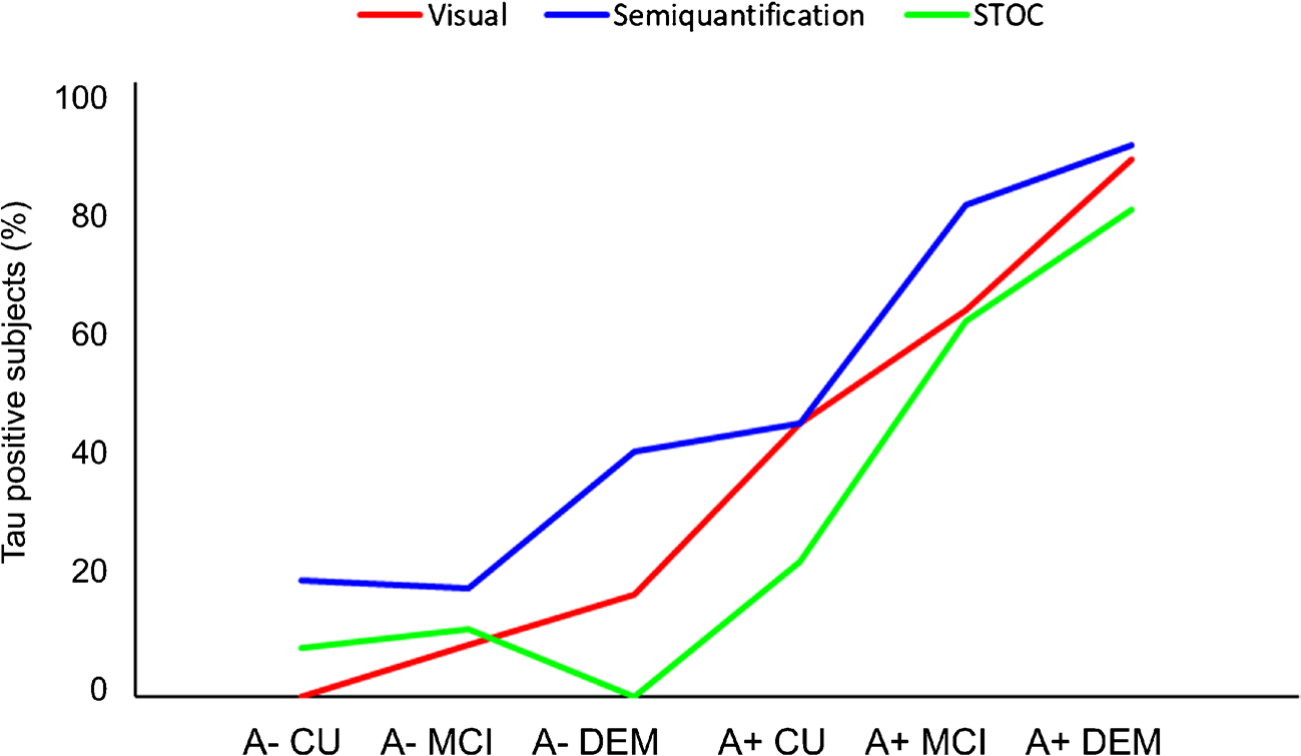
Distribution of tau-positive patients by modality, clinical diagnosis, and amyloid beta status. Tau positivity is represented as percentages. Results based on global SUVR, visual Braak staging and STOC staging are shown in blue, red, and green, respectively. Abbreviations: CU, cognitively unimpaired; MCI, mild cognitive impairment; DEM, dementia; A, amyloid; STOC, Simplified Temporo-Occipital Classification

When we considered clinical stages, we found a substantial rise in tau load as measured by global SUVR from CU to DEM (*p*<0.001) (Table 3). Tau positivity increased regardless of the tau assessment’s methods (Table 3). Using global SUVR resulted in the highest T+ rates, for all clinical diagnoses, independently from Aβ status, with T+ rate progressively increasing from CU to MCI to DEM in Aβ-positive participants. Visually defined T+ rates progressively increased from CU to MCI to DEM in Aβ-positive participants, with the lowest T+ rates observed in Aβ-negative participants, reaching zero in Aβ-negative CU. STOC-based T+ rates fell between visual- and global SUVR-based rates in CU, were similar to visual assessment in MCI, and exhibited lower rates in DEM, ultimately reaching zero T+ in Aβ-negative CU (Fig. 1 and Table 3).

**Table 3:** Summary of all -PET status and stages by modality

	CU	MCI	DEM	*p* value
Tau Global-SUVR ^*#*^	1.2(0.14)	1.3(0.27)	1.6(0.38)	<0.001
T+/T− Global-SUVR ^*#*^	15/50	67/54	33/8	<0.001
T+/T− Visual	7/65	56/70	34/13	<0.001
T+/T− STOC ^*#*^	10/55	53/68	28/13	<0.001
Visual Braak				<0.001
0	57	53	8	
I-III	8	15	4	
IV	0	8	3	
V	6	39	21	
VI	1	9	10	
Non-AD	0	2	1	
STOC stages ^*#*^				<0.001
0	45	41	5	
1	10	27	8	
2	0	25	7	
3	5	17	9	
4	4	9	12	
Atypical	1	2	0	

*Abbreviations: CU* cognitively unimpaired, *MCI* mild cognitive impairment, *DEM* dementia, *STOC* Simplified Temporo-Occipital Classification, *SUVR* standardized uptake value ratio, *T* tau

^#^Semi-quantitative measures are obtained in 227 participants instead of the total sample of 245

**Fig. 2:**
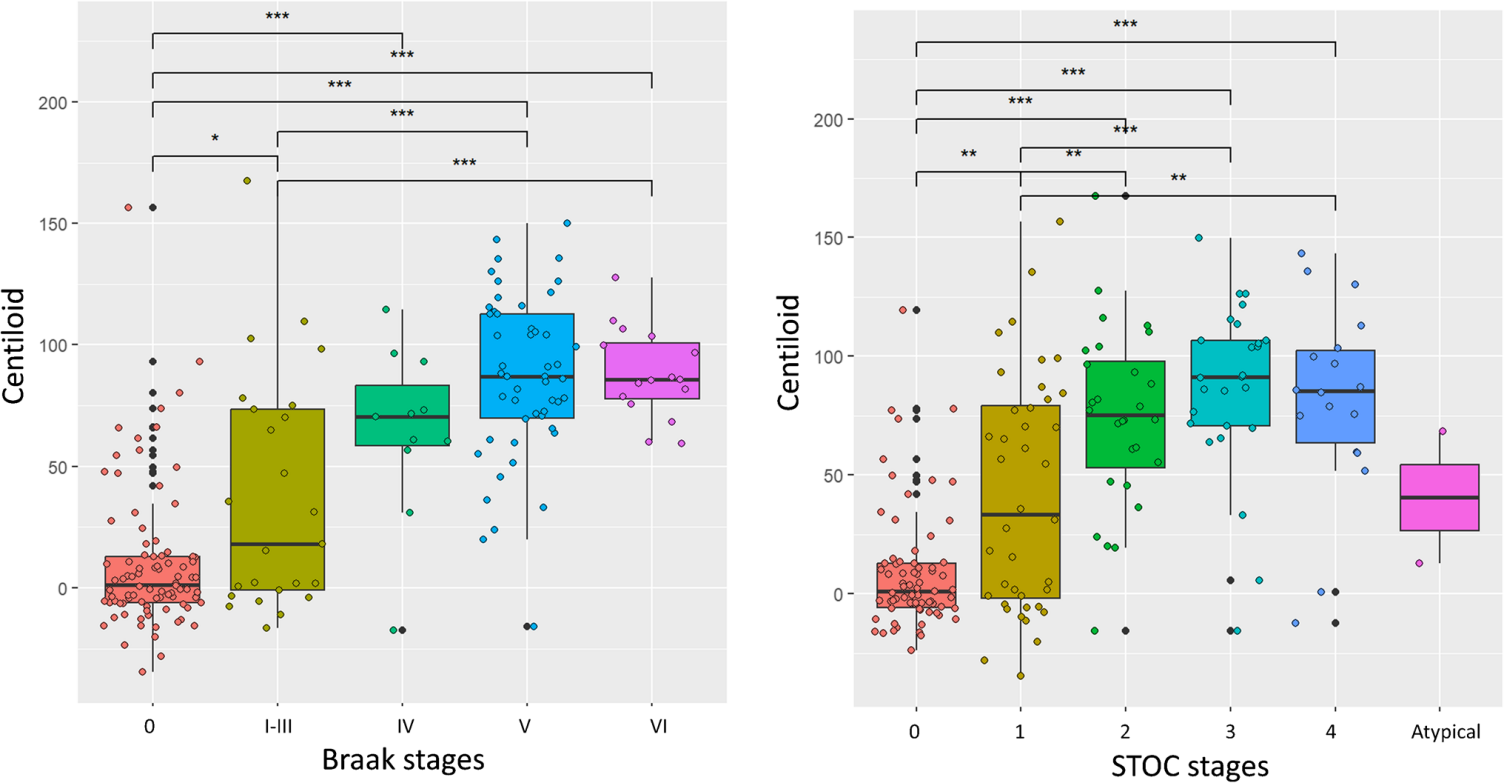
Association between Amyloid distribution (Centiloid) and Braak visual stages and STOC stages. * indicates significant p-values on the top and corresponds to post hoc tests following significant Kruskal-Wallis test. Abbreviations: STOC, Simplified Temporo-Occipital Classification

When considering amyloid burden (centiloid), we found a progressively rising value from visually defined Braak stage 0 to stage V/VI (*p*<0.01) (Fig. 2), similarly observed with the semi-quantitative STOC classification, with the highest values for stages 3 and 4 (Fig. 2).

**Fig. 3:**
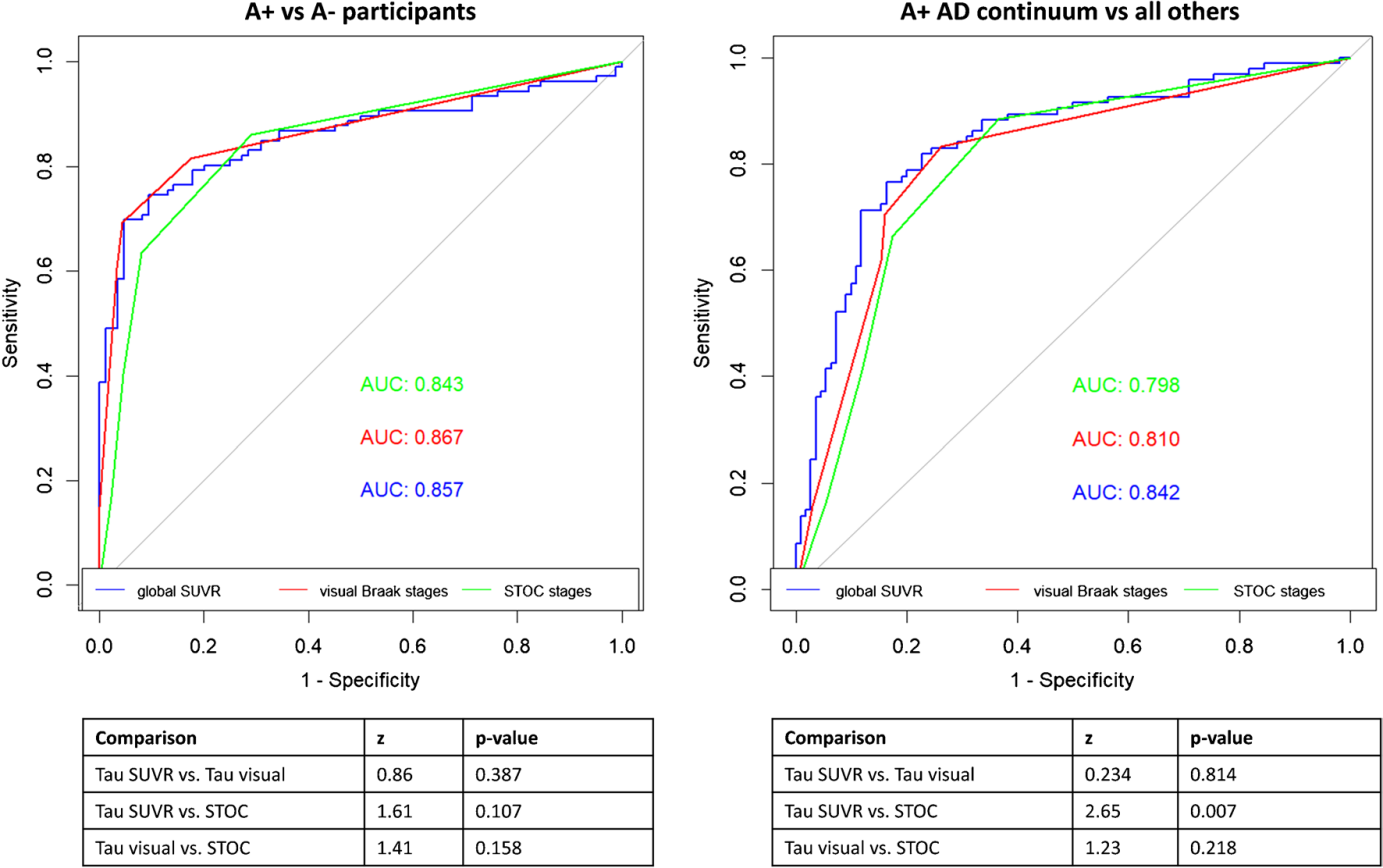
Discriminative performance of different tau assessment’s modalities. ROC testing on the ability of different modalities to distinguish Aβ-negative from Aβ-positive cases (A), and Aβ-positive MCI and DEM cases from all other clinical cases (B). AUCs for global SUVR, visual Braak staging and STOC staging are shown in blue, red, and green, respectively. Abbreviations: AUC, area under the curve; MCI, mild cognitive impairment; DEM, dementia; STOC, Simplified Temporo-Occipital Classification; SUVR, standardized uptake value ratio

To evaluate the ability of different modalities to distinguish Aβ-negative from Aβ-positive cases, ROC analyses were conducted. The area under the curve (AUC) was 0.86 for global SUVR, 0.87 for visual assessment of Braak stages, and 0.84 for STOC stages. The De Long test did not find significant differences between modalities (*p*>0.05) (Fig. 3). Similarly, ROC analyses were performed to assess the ability to distinguish Aβ-positive MCI and DEM cases from all other clinical cases. The area under the ROC curve was 0.84 for global SUVR, 0.81 for the visual assessment of Braak stages, and 0.80 for STOC stages. The De Long test found a significant difference only between the global SUVR and the STOC stages AUCs (*p*=0.007) (Fig. 3).

### Longitudinal cognitive trajectories

**Fig. 4:**
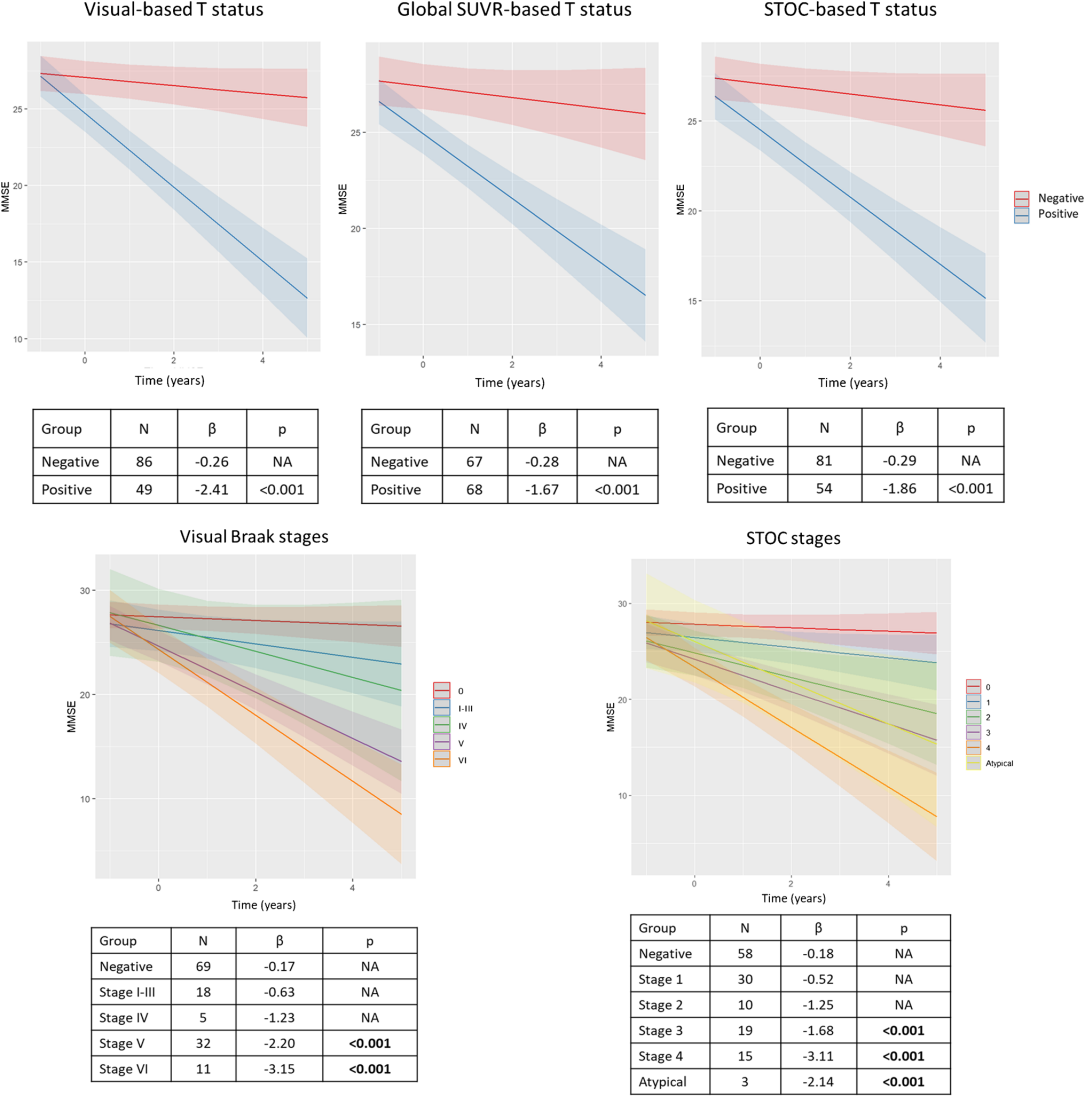
Longitudinal results. The upper part of the figure shows different cognitive trajectories of MMSE scores over time in the different modalities (visual, global SUVR, and STOC). The lower panel shows different cognitive trajectories of MMSE scores over time in the different stages, Braak Visual assessment on the left and STOC classification on the right. Abbreviations: STOC, Simplified Temporo-Occipital Classification; SUVR, standardized uptake value ratio; T, tau

The findings obtained with linear mixed effect models showed comparable cognitive trajectories of T+ groups over time, independently from tau assessment method. Specifically, visually defined T+, STOC-based T+, and global SUVR-based T+ subjects displayed a faster decline in MMSE scores over time when compared to their T− counterparts. The standardized *β* values for the interaction with time (years) were −2.41 (*p*<0.001) for the visual-based T+ group, −1.67 (*p*<0.001) for the STOC-based T+ group, and −1.86 (*p*<0.001) for the SUVR-based T+ group (Fig. 4).

When considering stages (visual Braak and STOC) instead of the binary T status, individuals in Braak stages V/VI and STOC stages 3/4 and atypical displayed a significantly steeper cognitive decline over time compared to negative subjects (Braak 0 and STOC 0). Standardized *β* values for Braak stages V/VI were −2.20/−3.15 (*p*<0.001), whereas standardized *β* values for STOC stages 3/4 were −1.68/−3.11 and −2.14 for the atypical pattern (*p*<0.001) (Fig. 4).

## Discussion

The present study addressed the need for a reliable approach for the visual interpretation of [^18^F]flortaucipir PET scans for the classification of subjects evaluated in a memory clinic for diagnostic and prognostic purposes. The high inter-rater agreement we obtained supports the reliability of our visual assessment method that strongly correlated with SUVR quantification, clinical stages, and Aβ loads. This visual interpretation assessment, tested in a large memory clinic sample, showed diagnostic and prognostic abilities comparable to SUVR-based quantification methods, with a similar performance in classifying subjects and predicting the risk of cognitive decline.

First, we found good inter-rater agreement among three nuclear medicine physicians with different levels of expertise in the visual classification of subjects and interpretation of tau Braak stages (*k*>0.68). Our inter-rater reliability values were similar to the results obtained in previous studies [6, 12, 14], despite the use of non-identical visual rating schemes. Moreover, the agreement observed here was consistent regardless of the raters’ level of expertise, indicating that visual interpretation of [^18^F]flortaucipir PET scans can be reliably performed also by less experienced raters, after a short training. This finding is crucial for the potential broader clinical application of [^18^F]flortaucipir PET, as it suggests that visual reading could be employed at a large scale, making the technique more accessible.

Few previous studies compared visual and semi-quantitative assessments for [^18^F]flortaucipir PET [12–14], reporting a good agreement between visual and semi-quantitative methods in defining positive and negative scans regardless of different diagnostic categories. Our results complemented this evidence expanding the validated visual method by introducing a finer staging approach. Indeed, our study showed a good agreement between visual and semi-quantitative ratings also in defining tau stages (*k*_weighted_ =0.73). This compelling agreement suggests that visual and semi-quantitative approaches yield consistent outcomes despite their distinct methodologies. In line with previous studies [12–14], we also confirmed a strong association between visual ratings and clinical and Aβ status.

We found progressively rising Aβ centiloid values from Braak stage 0 to stage V/VI, as defined with the visual classification, as well as with the semi-quantitative STOC classification with the highest values for stages 3 and 4 (Fig. 2). When we consider both Aβ status and clinical diagnosis, we observed a strong agreement between T+ by visual and quantitative assessments among Aβ-positive DEM patients (Fig. 1), consistent with the high uptake typically observed in this group [26]. However, we did encounter also “false positive” [^18^F]flortaucipir PET scans in Aβ-negative individuals, particularly when using global SUVR and STOC classifications. They could be explained by off-target binding of the tracer or they might represent cases of neurofibrillary pathology without Aβ, such as primary age-related tauopathy (PART). However, the unavailability of autopsy data hampers our ability to determine the nature of these [^18^F]flortaucipir PET findings. The main obstacle encountered when evaluating an [^18^F]flortaucipir PET relates to the presence of off-target binding, which displays considerable variability in severity and location across individuals, making the interpretation of [^18^F]flortaucipir PET results more intricate [10]. This off-target signal is thought to be influenced by several factors, including neuromelanin, brain hemorrhagic lesions, and the widespread distribution of the enzymes monoamine oxidase (MAO)-A and MAO-B throughout the brain in both neurons and glia [27, 28]. Mild [^18^F]flortaucipir PET signal in subcortical white matter has been reported in patients with frontotemporal lobar dementia (FTLD)-tau related and in anterior temporal lobes in patients with semantic variant of primary progressive aphasia (svPPA) [29]. The underlying reasons for tracer accumulation in this context are not yet understood, but it might represent another source of off-target binding. Among Aβ-negative subjects the rate of positive cases was lower using the visual classification as compared to the two semi-quantitative methods (Fig. 1) suggesting a higher specificity of visual rating. This is supported by the observation of follow-up data in our cohort, which is available for 11 out of the 14 subjects classified as T+ semi-quantitatively and T− visually: all are stable after 3 years.

The higher specificity of visual rating could be explained by the raters’ higher ability to correctly distinguish off-target binding and cortical uptake.

Differently from us, previous studies have reported a higher rate of T+ across all different diagnoses and Aβ-status when using the visual approach compared to SUVR-based classification [13]. This disparity may be explained by the uncertain significance of mild temporal binding alone. Indeed, in our classification an isolated mesial temporal uptake was considered it as T-, following the FDA-approved visual reading scheme [6], whereas Provost and colleagues [13] considered subjects exhibiting binding restricted to the medial and/or anterior regions as T+, possibly reflecting early AD pathology, to increase the sensitivity at the expense of specificity.

Visual- and SUVR-based ratings allowed a comparable good differentiation of subjects in the AD spectrum from other participants (AUCs ranging between 0.84 and 0.87) (Fig. 3), further confirming the clinical applicability of visual rating. The AUC values for the visual rating in our cohort are slightly lower compared to values reported by Sonni and colleagues [12] (0.81 versus 0.92), likely due to differences in the two visual rating schemes. Indeed, our rating relies more on tau stages instead of AD and non-AD patterns.

Lastly, our study investigated the prognostic performance of visual and semi-quantitative assessments by studying longitudinal cognitive trajectories of individuals classified as T+. All three strategies identified T+ subjects with a faster cognitive decline as compared to T− subjects in a 2-year follow-up period (Fig. 4). Although the standardized *β* values for the interaction with time in years were remarkably consistent across T+ groups defined by different modalities, the highest value was found for the visual-based T+ group (−2.41, *p*<0.001) (Fig. 4). Given the established prognostic value of tau-PET, overperforming other biomarkers [8, 30, 31], the similar ability of visual and semi-quantitative approaches in discriminating cognitive decliners, demonstrated here, is crucial for the implementation of tau-PET in clinical practice. Our results are in line with recent evidence [14] of the association between a positive tau-PET visual read and a steeper cognitive decline in CU and AD. Moreover, differently from the previous study [14], we also considered tau stages rather than binary T status. Individuals with advanced AD-pattern (visual-Braak stages V/VI and STOC stages 3/4) exhibited a significantly faster and steeper cognitive decline when compared to subjects without significant tau accumulation. These data highlight the potential prognostic value of visual reads and semi-quantitative measures in predicting cognitive decline due to AD, without a significative difference between methods, and appear to support the hypothesis that advanced AD patterns would be more informative for predicting participants’ near-term clinical progression than other patterns [16, 32].

Our study has some limitations. First, [^18^F]flortaucipir shows considerable off-target binding in the hippocampus and basal ganglia, which may confound the assessment of tau pathology [27, 28, 33]. Despite our effort to recognize regions affected by off-target binding, influences of unspecific binding remain possible, hence our findings await further replication with second-generation tau-PET tracers (i.e., radiotracers with a better off-target binding profile [9]). Second, we evaluated our subjects at a relatively short follow-up period and the study did not include a follow-up [^18^F]flortaucipir PET scan, which could provide more comprehensive information about the progression of tau pathology and its relationship to cognitive decline over time. Third, we used MMSE as a measure of cognitive decline and we are aware that MMSE is a global measure characterized by a ceiling effect, less sensitive than other tests or test combinations [34, 35]. Cognitive scales commonly used in clinical trials, such as Consortium to Establish a Registry for Alzheimer’s Disease neuropsychological assessment battery (CERAD-NAB), Alzheimer's Disease Assessment Scale (ADAS) and Clinical Dementia Rating (CDR)-Sum of Boxes were not measured for our sample, and the PACC measure was available only in a subgroup.

Lastly, we lack an autopsy-based gold standard, which would be the ideal reference to evaluate the performance of the proposed visual approach.

A major strength of this study is the large number of subjects recruited from a memory clinic setting that enhances the clinical applicability of our results.

## Conclusion

The visual rating scheme tested here showed strong associations with well-established semi-quantitative measures, clinical stages, and Aβ status in our memory clinic cohort. Moreover, visual assessment exhibited a performance comparable to semi-quantitative indices in identifying AD subjects and in predicting cognitive decline. These findings together with the reproducibility of the visual method supported by the strong inter-rater reliability support its readiness for a routine implementation in a clinical setting.

### Supplementary Information

Below is the link to the electronic supplementary material.

Supplementary file1 (DOCX 180 KB)

## Data Availability

The datasets generated during and/or analyzed during the current study are available from the corresponding author upon reasonable request.
