# Supplementary material for: A comparison of visual assessment and semi-quantification for the diagnostic and prognostic use of [18F]flortaucipir PET in a memory clinic cohort
Source: Eur J Nucl Med Mol Imaging. 2024 Jan 6;51(6):1639–50. doi: 10.1007/s00259-023-06583-9 (PMC11041710; doi:10.1007/s00259-023-06583-9)
Supplement: Supplementary file 1 — Supplementary file1 (DOCX 180 KB) [file 259_2023_6583_MOESM1_ESM.docx]

**Supplementary Materials**

**S1. Longitudinal Analysis Considering PACC**

Preclinical Alzheimer’s Cognitive Composite (PACC) is a sensitive composite measure of multi-domain neuropsychological tests developed specifically to detect cognitive changes in preclinical stages ^1^. The PACC is derived from the combination of tests capturing episodic memory, executive function, semantic memory, and global cognition ^2^. In our cohort we specifically included four tests: Free and Cued Selective Reminding Test (FCSRT) delayed recall, WAIS-R Digit-Symbol, Fruits fluency, and Mini-Mental State Examination (MMSE), respectively. The episodic memory test was given double weight and thus accounted for 40% of the score ^2^. The total score of PACC was calculated as the sum of the normalized z-scores of these four measures. This means that individual tests were z-transformed using the baseline test scores of volunteers who underwent those tests at the Geneva Memory Centre as the reference group and then averaged to obtain a composite z-score. We could calculate the PACC score for 83 subjects in our sample at baseline and a subsample of 39 subjects also has follow-up measure. Linear mixed-effects models to assess the prognostic value of tau status were applied including random intercepts and slopes, with longitudinal PACC scores as the dependent variable, and age and gender as covariates of noninterest. The longitudinal results showed comparable cognitive trajectories as measured by PACC of T+ groups over time independently from tau assessment methods (**Figure S1**). T+ groups showed a steeper cognitive decline compared to T- groups for all strategies, however, we found a significant difference in cognitive trajectories between groups only with the visual-based T status, differently from results obtained with MMSE as a measure of cognitive decline (**Figure 4**). Results based on PACC likely suffer from a limited sensitivity linked to the small sample size.


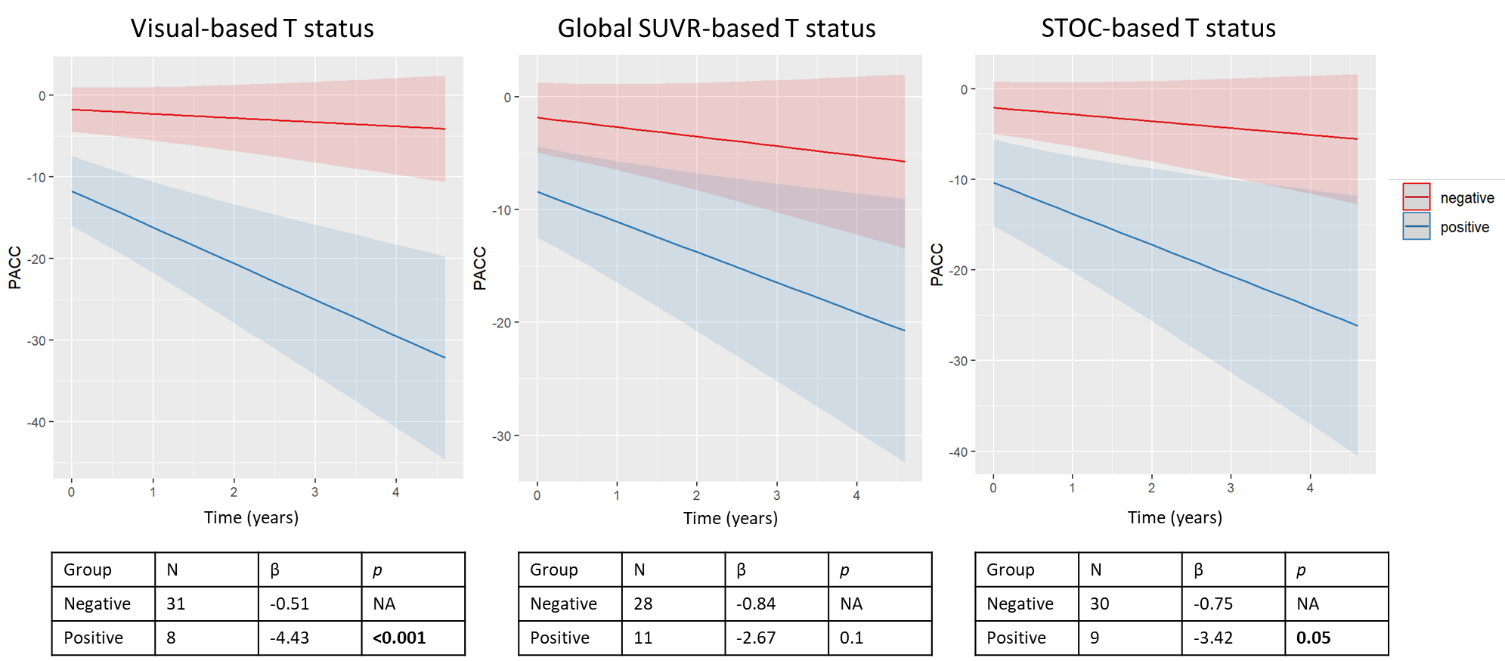


**Figure S1. Longitudinal results with PACC.** The figure shows different cognitive trajectories of PACC z-scores over time in the different modalities (visual, global SUVr and STOC). Abbreviations: STOC, Simplified Temporo-Occipital Classification; SUVR, standardized uptake value ratio; T, tau.
